# Adjusting the RAPID score with 2 additional variables significantly increases its predictive value in patients with empyema

**DOI:** 10.1038/s41598-023-29946-6

**Published:** 2023-02-24

**Authors:** B. O. Stüben, G. A. Plitzko, F. Urban, H. Kölzer, M. Kemper, J. Wakker, J. R. Izbicki, K. Bachmann

**Affiliations:** Department of General, Visceral and Thoracic Surgery, University Medical Center Hamburg-Eppendorf, Martinistraße 52, 20246 Hamburg, Germany

**Keywords:** Prognosis, Respiratory tract diseases, Risk factors

## Abstract

Pleural empyema is a serious condition leading to a significant burden on health care systems due to protracted hospitalisations. Treatment ranges from non-surgical interventions such as antibiotic therapy and chest tube placement to thoracoscopic or open surgery. Various risk factors which impact outcomes have been investigated. The RAPID (renal, age, purulence, infection source, and dietary factors) score is a clinical risk score which identifies patients at risk of death and may be used to formulate individual treatment strategies accordingly. All patients undergoing surgical interventions for empyema at a major tertiary medical centre in Germany from 2017 to 2020 were analysed. The aim was to identify perioperative risk factors which significantly impact treatment outcomes but are currently not included in the RAPID score. 245 patients with pleural empyema surgically treated at the Department of General, Visceral and Thoracic Surgery at the University Medical Centre, Hamburg, Germany (admitted from January 2017 to April 2020) were retrospectively analysed. All patients which received either minimally invasive or open thoracic surgery were included. Epidemiological as well as perioperative data was analysed to identify risk factors which impact long-term overall outcomes. 90-day mortality rate was the primary endpoint. The mean age was 59.4 years with a bimodal distribution. There was a male predominance across the cohort (71.4% compared to 28.6%), with no significant differences across ages below or above 60 years. 53 (21.6%) patients died within the first 90 days. Diabetes type 1 and 2, renal replacement therapy, immunosuppression, postoperative bleeding, intraoperative transfusion as well as microbiologically confirmed bacterial invasion of the pleura all led to higher mortality rates. Higher RAPID scores accurately predicted higher 90-day mortality rates. Modifying the RAPID score by adding the comorbidities diabetes and renal replacement therapy significantly increased the predictive value of the score. We demonstrated various perioperative and patient related risk-factors not included in the RAPID score which negatively impact postoperative outcome in patients receiving surgical treatment for pleural empyema. These should be taken into consideration when deciding on the best course of treatment. If confirmed in a prospective study including non-surgical patients with a significantly larger cohort, it may be worth considering expanding the RAPID score to include these.

## Introduction

Empyema can form in the pleural cavity because of pneumonia, thoracic surgery or as a complication of blunt or penetrating chest wall trauma. Oesophageal rupture and bronchogenic carcinoma are less common causes. Regardless of the etiology, the invasion of the sterile pleural space by bacteria always precedes the development of pleural empyema. Following the widespread use of antibiotics, the incidence of pleural empyema had been decreasing. However, in the last 20 years a continuous rise of cases and an increase in mortality rates has been reported by several authors^1–4^. Dramatically, a study from the UK showed that in over 60-year-olds, empyema related episodes doubled over a 10-year period from 2008 to 2018^5^. There is a nearly two-fold predominance in males to females^6^. Multimorbidity is a risk factor, with multimorbid patients twice as likely to develop pleural empyema^6^. Treatment options differ according to stage of disease as well as patient comorbidities. In the presence of infected fluid in the pleural space, antibiotic therapy may be considered as the cornerstone of treatment, while pus in the pleural space requires additional chest tube drainage. Surgical therapy is necessary when irrigation of the pleural cavity via the placement of a chest drain is complicated by fibrous septations, and recent therapy recommendations lean towards earlier intervention^3,7^. The goal of surgical therapy is the complete irrigation of the pleural cavity and re-expansion of the lung. The surgical approach is dictated by the condition of the pleural space as well as the condition of the patient. If single-lung ventilation is tolerated, video assisted thoracoscopic surgery (VATS) should be attempted^8^. However, open surgery is often necessary for patients with advanced empyema where thick tissue membranes are present and decortication, necessary for complete lung expansion, is often difficult. In cases of incomplete postoperative lung re-expansion, tissue flaps may be warranted to fill the pleural cavity to prevent reformation of fluid collections. Open thoracic window treatment (OTW) is indicated only in patients which are unfit to undergo decortication or tissue flap placement^8^ as a rescue procedure. Intrathoracic negative pressure wound therapy (NPWT) is a relatively new treatment option for pleural empyema. NPWT is now widely recommended for the closure of OTW wounds or at least volume reduction^7^.

With the aim of identifying those at high risk of death and improving therapeutic algorithms, the RAPID score (renal, age, purulence, infection source, and dietary factors) was developed and is one of the newer but widely used clinical risk scores^9^. Based on urea levels, age, purulence of pleural fluid, infection source (community vs. hospital-acquired) and albumin levels, risk categories (low, medium, high) are allocated. Mortality rates of 30–50% have been described in the high-risk group^9^.

The aim of our study was the validation of the predictive power of the RAPID score in a surgical cohort, and to identify perioperative risk factors which significantly impact treatment outcomes but are currently not included in the RAPID score.

## Methods

### Data collection

All patients undergoing surgical therapy for pleural empyema at the Department of General, Visceral and Thoracic Surgery at the University Medical Centre, Hamburg, Germany from January 2017 to April 2020 were analysed. The study comprised 245 patients. Patients received either VATS or open surgery.

The medical records of all patients participating were analysed retrospectively from our prospective database. Preoperative demographic data, comorbidities, ASA (American Society of Anaesthesiologists) score and the RAPID score of each patient was recorded. Microbiological results as well as antibiotic sensitivity testing was documented. Surgical technique as well as chest drain type and size were investigated. With the aim of analysing and validating the predictive value of the RAPID score in a surgical cohort only, we then investigated the effect of the RAPID score on the 90-day mortality-rate as the primary endpoint.

After having identified additional risk factors which had a statistically significant impact on 90-day overall mortality, we added these to the RAPID score to form an adjusted RAPID score. We then compared the predictive value of the original RAPID score with the adjusted RAPID score regarding 90-day mortality.

### Statistical analysis

Statistical analysis was carried out using *IBM SPSS ver. 24 (Armonk, N.Y., USA)*. The Wilcoxon rank test was used for continuous variables. The χ2 test and Fischer’s test were employed for categorical variables. A *p-value* ≤ of 0.05 was considered significant. Parameters which were shown to have a statistically significant impact on 90-day mortality rates in the univariate analysis were then tested in a multivariate COX regression analysis to detect parameters independently associated with an increase in 90-day mortality rates. Patients without each individual comorbidity served as reference group.

The results were presented as hazard ratios with 95% CI. All comparisons were two‐tailed.

### Ethics approval and consent to participate

The study was conducted according to the guidelines of the Declaration of Helsinki, and was approved by the Ethics Committee of Hamburg, Germany (protocol code: PV3548).

## Results

### Baseline demographics, comorbidities, blood/pleural analysis and cause of empyema

A total of 245 patients were surgically treated at our institution for pleural empyema from January 2017 to April 2020. 20 patients were lost to follow up and therefore were not included in final analysis.

The mean age was 59.4 (± 15.7) years with a bimodal distribution. There was a male predominance across the cohort (69.4% compared to 30.6%). Mean (standard deviation, SD) BMI (Body Mass Index) was 25.3 ( ±) 5.5 kg (Table 1). 191 patients received an open thoracotomy, while 53 patients received VATS. The leading cause for empyema was pneumonia (62.0%). Less common were lung cancer (8.6%), blunt trauma (3.7%) and penetrating trauma (1.2%). Among the analysed patients, hypertension (46.5%), atrial fibrillation (25.4%), renal replacement therapy (RRT) (18.0%) and diabetes mellitus (17.2%) were the most prevalent comorbidities. Smoking and alcohol problems were found in 38.4% and 33.4%, respectively. 25.4% of the patients had a pre-existing immunosuppression.

**Table 1:** Univariate analysis of demographic and clinical data.

	Available data (n)	Overall (n = 225)	Survived 90 days (n = 174)	Died within 90 day (n = 51)	*p*-value
Age, years^#^	225	62 (51, 70)	61 (49,70)	65 (57, 73)	**0.045**
Gender	225				0.10
Male^#^		160 (71)	119 (68)	41 (80)	
Female^#^		65 (29)	55 (32)	10 (20)	
Median BMI, kg/m^#^	225	25 (22, 28)	25 (22, 28)	26 (22, 29)	0.59
Comorbidities					
Asthma^#^	225	8 (3.6)	7 (4)	1 (2)	0.69
OSAS^#^	225	8 (3.6)	5 (2.9)	3 (5.9)	0.40
COPD^#^	223	36 (16)	28 (16)	8 (16)	0.90
Diabetes^#^	224	39 (17)	22 (13)	17 (33)	** < 0.001**
Atrial fibrillation^#^	224	60 (27)	41 (24)	19 (37)	0.12
Hypertension^#^	225	101 (45)	72 (41)	29 (57)	0.05
Cardiac insufficiency^#^	225	34 (15)	23 (13)	10 (20)	0.20
RRT^#^	225	28 (12)	9 (5)	19 (37)	** < 0.001**
Liver cirrhosis^#^	48	8 (17)	6 (15)	2 (22)	0.60
Smoking^#^	127	91 (72)	70 (69)	21 (84)	0.13
Alcohol problems^#^	138	50 (36)	37 (34)	14 (46)	0.20
Therapeutic anticoagulation^#^	224	66 (29)	46 (27)	20 (39)	0.08
Immunosuppression^#^	224	57 (25)	37 (21)	20 (39)	**0.01**
Surgical procedure	225				0.13
Thoracotomy^#^		172 (76)	129 (74)	43 (84)	
VATS^#^		53 (24)	45 (26)	8 (16)	
Perioperative blood analysis					
WBC, G/L*	225	366 (235, 501)	393 (281, 518)	241(145, 370)	0.14
Haemoglobin (g/dL)*	225	9.6 (8.4, 10.9)	9.7 (8.6, 10.3)	8.6 (7.9, 10.3)	**0.002**
CRP (mg/L)^#^	225	131 (73, 181)	126 (68, 179)	146 (95, 196)	0.08
Urea(mg/dL)^#^	225	16 (10, 26)	15 (10, 24)	22 (11, 30)	**0.05**
Creatinine (mg/dL)^#^	225	0.83 (0.63–1.20)	0.80 (0.62, 1.00)	0.97 (0.73, 1.60)	**0.008**
Albumin (g/L)*	151	17 (14, 21)	18 (15, 22)	15 (10, 19)	**0.003**
Blood culture^#^	219	176 (80)	129 (76)	47 (96)	**0.002**
Perioperative factors					
Bacterial invasion of the pleural space^#^	221	125 (57)	85 (50)	40 (80)	** < 0.001**
Postoperative bleeding^#^	224	27 (12)	10 (6)	17 (33)	** < 0.001**
Perioperative blood transfusion^#^	224	96 (43)	61 (35)	35 (69)	** < 0.001**

BMI, Body mass index, COPD, Chronic obstructive pulmonary disease, CRP, C-reactive protein, LDH, Lactate dehydrogenase, OSAS, Obstructive sleep apnoea syndrome, PTT, Partial thromboplastin time, RRT, Renal replacement therapy, WBC, White blood cell count.

^#^Data presented as median (interquartile range.

*Data presented as median or n (%).

Statistical significance was tested using χ2 test for categorical variables and Wilcoxon-rank test for continuous variables. Bold *p*-values are of statistical significance (≤ 0.05).

### Univariate analysis

Higher age and urea as well as lower albumin levels, all of which are components of the RAPID score, had a significant impact on the 90-day mortality. Positive blood cultures and bacteria in the pleural fluid were also associated with increased mortality.

Diabetes, RRT and immunosuppression were comorbidities currently not included in the RAPID score which led to a significantly higher mortality.

Postoperative bleeding (*p* < 0.001) and perioperative blood transfusion (*p* = 0.041) led to significantly higher mortality rates in patients surgically treated for pleural empyema.

Importantly, the type of surgical procedure (VATS or thoracotomy) did not have a significant impact.

The results of the univariate analysis are shown in Table 1.

### Multivariate analysis

The comorbidities which were identified as having a significant impact on 90-day mortality rates in the univariate analysis were then included in a multivariate analysis. Of the perioperative factors, positive blood cultures and bacterial invasion of the pleural fluid were included in the analysis. Postoperative bleeding and perioperative blood transfusion were not included in further analyses as these factors are always observed post-hoc and adding them to a preoperative risk score is not clinically applicable.

Multivariate Cox regression model confirmed statistical significance for RRT (HR = 2.47, 95% CI1.32–4.62, p = 0.005), Diabetes (HR = 2.00, 95% CI 1.07–3.76, *p* = 0.031) and bacterial invasion of the pleural space (HR = 5.80, 95% CI 1.18–4.89, *p* = 0.016) as predictive factors for 90-day mortality. There was a trend of increased 90-day mortality in patients with immunosuppression (HR = 1.51, 95% CI 0.84–2.74, *p* = 0.170) and positive blood culture (HR = 3.72, 95% CI 0.88–15.86, *p* = 0.075) that did not reach statistical significance. These results are presented in Table 2.

**Table 2:** Multivariate COX regression analysis.

	ß-coefficient	Standard error	Wald × 2	*p*-value	HR	95% CI
RRT	0.41	0.32	7.99	**0.005**	2.47	1.32–4.62
Immunosuppression	0.41	0.30	1.88	0.170	1.51	0.84–2.74
Diabetes	0.69	0.32	4.68	**0.031**	2.00	1.07–3.76
Positive blood culture	1.32	0.74	3.17	0.075	3.72	0.88–15.86
Bacterial invasion of the pleural space	0.88	0.36	5.80	**0.016**	2.40	1.18–4.89

RRT, renal replacement therapy.

Bold *p*-values are of statistical significance (≤ 0.05).

### RAPID Score

**Figure 1:**
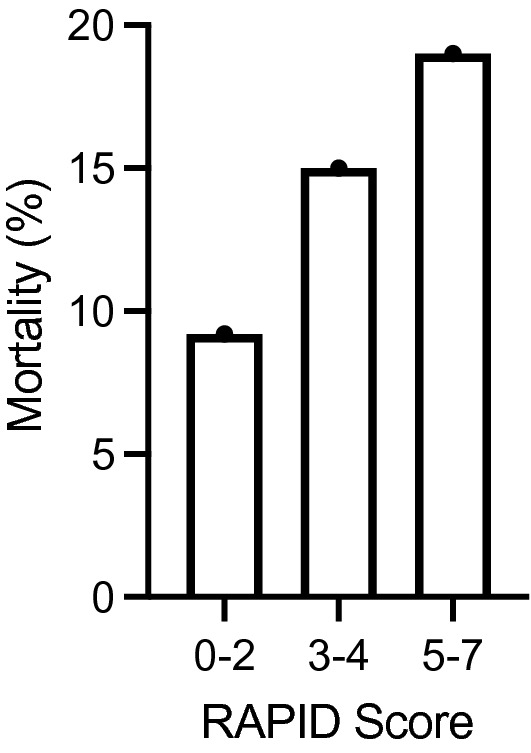
Effect of the RAPID score on 90-day mortality rate (%).

In line with previous data, higher RAPID Scores showed a positive correlation with 90-day mortality in our study (Fig. 1). Higher RAPID scores significantly increased 90-day mortality rates in patients surgically treated for pleural empyema in our study (*p* = 0.002). The single components of the RAPID score (age, urea, albumin) all showed a significant impact on 90-day mortality.

### Adjusted RAPID Score

After validating the predictive value of the RAPID score in our surgical cohort, diabetes and RRT were then added to the RAPID score to form an adjusted RAPID score. Positive blood cultures and bacterial invasion of the pleura fluid were not added, as the results of these tests may take up to 5 days following admission until available and therefore do not appear useful as factors in a prognostic tool which should be easy and quick to evaluate.

Table 3 shows how the adjusted RAPID score was calculated.

**Table 3:** Components of the adjusted RAPID score and definition of risk categories.

Parameter	Measure	Score
Renal	Urea (mM)	
	< 5	0
	5–8	1
	> 8	2
Age	Years	
	< 50	0
	50–70	1
	> 70	2
Purulence of pleural fluid		
	Purulent	0
	Nonpurulent	1
Infection source		
	Community acquired	0
	Hospital acquired	1
Dietary factors	Albumin, g/L	
	≥ 27	0
	< 27	1
Diabetes		
	No	0
	Yes	1
RRT		
	No	0
	Yes	1
Adjusted RAPID score Risk categories		
	Score 0–2	Low risk
	Score 3–4	Intermediate risk
	Score 5–7	High risk
	Score 8–9	Very high risk

RRT, renal replacement therapy.

**Figure 2:**
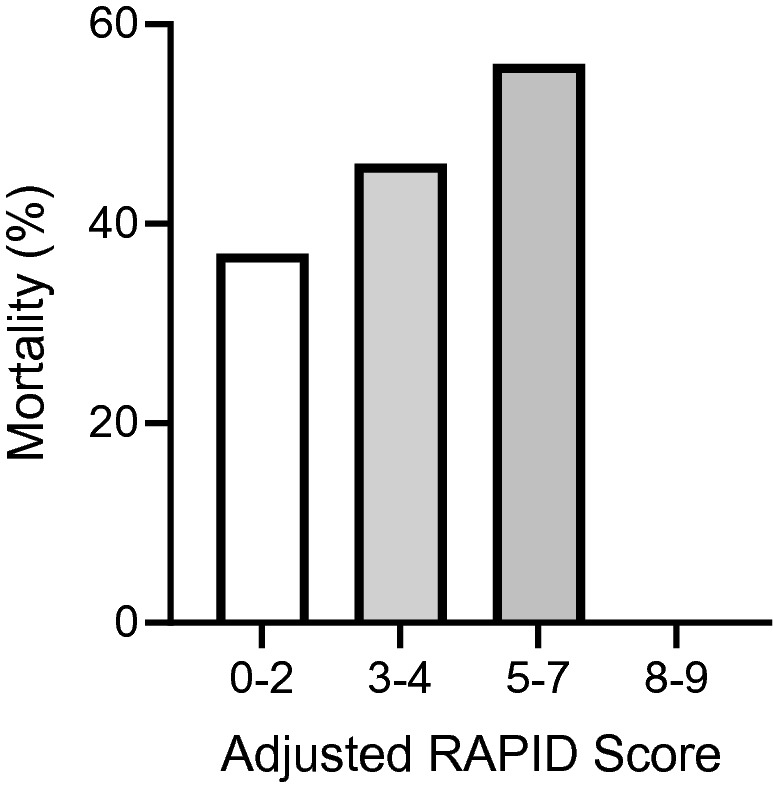
Effect of the adjusted RAPID score on 90-day mortality rate (%).

An increasing adjusted RAPID-score led to higher 90-day mortality rates, as shown in Fig. 2. No patients reached a score of 8 or greater.

### Predictive value of additional clinical parameters compared with RAPID Score only

**Figure 3:**
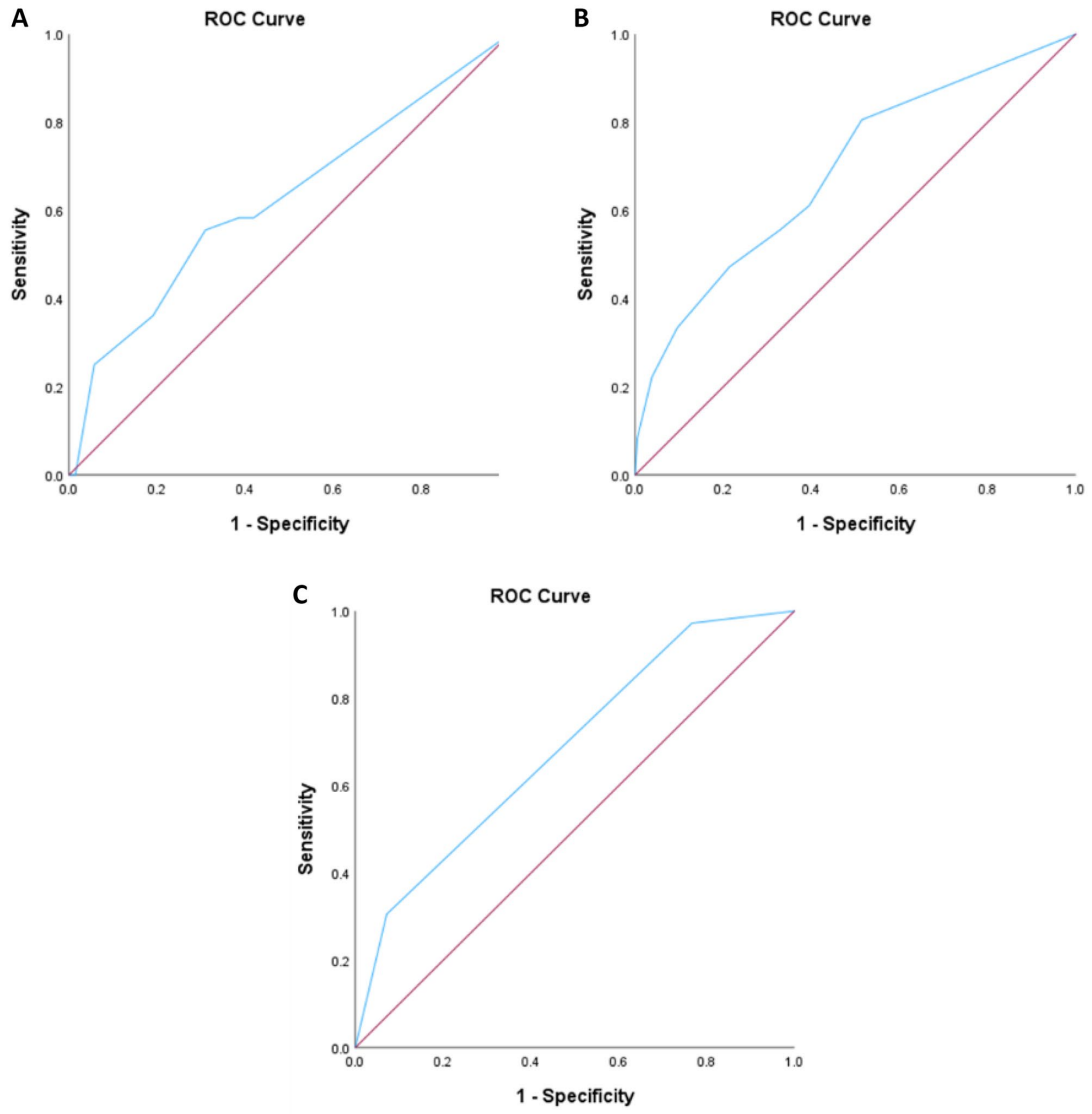
ROC curves of RAPID score (**A**), adjusted RAPID score (**B**) and Comorbidities (**C**).

In a ROC analysis, we assessed if the RAPID score accurately predicted 90-day mortality rates in a surgical cohort. In line with previous data, the RAPID score alone accurately predicted 90-day mortality rates with statistical significance Area Under the Curve (AUC) of 0.62 (95% CI 0.52–0.73, *p* = 0.018) (Fig. 3A).

We then tested the predictive value of the adjusted RAPID score in an ROC analysis. The adjusted RAPID score accurately predicted 90-day mortality rates (AUC: 0.69, 95% CI 0.59–0.79, *p* < 0.001) (Fig. 3B).

The predictive value of the adjusted RAPID score against the RAPID score was then compared. The adjusted RAPID score showed a significantly improved predictive value compared to the RAPID score (AUC difference 0.066, *p* = 0.002).

The AUC of the comorbidities (diabetes and RRT) alone was similar to the adjusted RAPID score (AUC 0.685, 95% CI 0.59–0.78, *p* < 0.001) (Fig. 3C). To rule out that the comorbidities alone have a better predictive value than the RAPID score, they were then tested in an ROC analysis against the RAPID score where no significant difference in AUC was observed (AUC difference 0.062, *p* = 0.281).

## Discussion

Pleural empyema is a condition leading to a significant burden on health care systems due to protracted hospitalisations. Mortality rates of up to 15% have been reported^10^. Treatment ranges from non-surgical interventions such as antibiotic therapy and chest tube placement to thoracoscopic or open surgery.

When deciding on the best course of treatment, it is often not clear which patients benefit from early surgical intervention and which would be better conservatively treated with antibiotics and drainage. For example, increasing mortality rates with increasing age could be due to biological factors or a surgeons’ reluctancy to treat pleural empyema surgically early on. Evidence that for stage 2 and 3 empyema surgical therapy has superior outcomes to tube thoracostomy alone could support this^11,12^.

There are various known patient-related risk factors which impair treatment success. Recently, attempts have been undertaken to improve patient triage and guide clinicians when deciding on the optimal treatment options for individual patients based on different risk factors. The RAPID Score is a clinical risk score which was developed to identify patients at higher risk of death at presentation and may be used to formulate individual treatment strategies accordingly^9^. In their work, the authors analysed baseline clinical and outcome data from the first Multicentre Intrapleural Sepsis Trial (MIST1) where patients with pleural empyema were treated with intrapleural streptokinase^13^. The derived model was then validated using baseline clinical and outcome data from the subsequent MIST2 trial^14^. It has since been further validated in a prospective observational cohort study^15^. The RAPID score proved to accurately predict 90-day mortality rates in non-surgical patients. The predictive accuracy for surgical cohorts is unclear.

In our study we were able to confirm that the single components of the RAPID score (age, urea, and albumin) accurately predicted 90-day mortality. Furthermore, ROC analysis of our data shows that the RAPID score does in fact accurately predict deteriorating patient outcomes, with higher scores correlating with increasing 90-day mortality rates.

We then investigated the comorbidities in our cohort and the effect these had on 90-day mortality. In the multivariate analysis, we identified diabetes and RRT as comorbidities which significantly impacted 90-day survival rates in our cohort. With the aim of increasing the predictive power of the RAPID score, we analysed the effect that adding these comorbidities to the score would have.

Adding these comorbidities to the RAPID score to create an adjusted RAPID score further improved the predictive value in terms of 90-day mortality rates for patients undergoing surgery for pleural empyema when performing ROC analysis. A comparison of the AUC of the ROC curves demonstrated that the adjusted RAPID score predicted 90-day mortality rates significantly better than the RAPID score alone. The comparison of the comorbidities alone and the RAPID score showed that while the trend showed the comorbidities to have a higher predictive value, however this did not reach significance.

The high predictive value of the comorbidities alone could be explained by the fact that these comorbidities may have a larger impact on the outcomes of patients undergoing surgery than patients being managed conservatively. The negative impact of diabetes and RRT for patients undergoing major non-cardiac surgery has been well documented^16–19^.

The presented study has several major limitations. Purulence of the pleural fluid defined by visual analysis by the clinician as used in the RAPID score and in our study is a subjective parameter, liable to inter-observer variability. There is a selection bias as only surgically treated patients were included. This could allow for the tentative conclusion that patients with more advanced empyema and generally sicker patients were included in our study compared to the initial RAPID score publication by Rahman et al.^9^, where only non-surgical patients were included. It remains to be seen if the adjusted RAPID score also accurately predicts outcomes in patients treated by antibiotics and drainage alone. The analysis of the AUC for the ROC curves does however allow for the statement that in the surgical subgroup, the predictive value of the adjusted RAPID score is significantly higher than the RAPID score. A study validating this score for non-surgical patients is necessary to allow for definite conclusions.

The small sample size and especially the small sample size of the different subgroups certainly limits the statistical power of our data. The retrospective nature of the analysis at a single centre further limits the strength of the results. Therefore, prospective studies with larger sample sizes, especially including non-surgical patients with the aim of validating the proposed adjustment of the RAPID score for all patients with pleural empyema are necessary to answer the question if the adjustment of the existing RAPID score as suggested by our data is in fact sensible and reliably predicts outcomes in patients with empyema.

## Conclusions

The RAPID score accurately predicts 90-day mortality in surgical patients. Diabetes and RRT significantly increase 90-day mortality in patients which undergo surgery for pleural empyema. We propose adjusting the RAPID score by including these comorbidities to improve patient triage and therapeutic decision making when patients initially present with pleural empyema. Prospective studies with larger sample sizes and with the aim of validating these findings for non-surgical patients are necessary.

## Data Availability

The data that support the findings of the study are available upon request from the corresponding author (B.-O.S.).
